# Association of Angiogenesis Gene Expression With Cancer Prognosis and Immunotherapy Efficacy

**DOI:** 10.3389/fcell.2022.805507

**Published:** 2022-01-26

**Authors:** Xin-yu Li, Wei-Ning Ma, Li-xin Su, Yuchen Shen, Liming Zhang, Yuhao Shao, Deming Wang, Zhenfeng Wang, Ming-Zhe Wen, Xi-tao Yang

**Affiliations:** ^1^ Department of Interventional Therapy, Shanghai Ninth People’s Hospital, Shanghai Jiao Tong University School of Medicine, Shanghai, China; ^2^ Department of Neurosurgery, Shanghai Ninth People’s Hospital, Shanghai Jiao Tong University School of Medicine, Shanghai, China; ^3^ Department of Pediatrics, Shanghai General Hospital, Shanghai Jiao Tong University School of Medicine, Shanghai, China

**Keywords:** angiogenesis, pan-cancer, prognosis, methylation, gene expression

## Abstract

**Background:** Several new blood vessels are formed during the process of tumor development. These new blood vessels provide nutrients and water for tumour growth, while spreading tumour cells to distant areas and forming new metastases in different parts of the body. The available evidence suggests that tumour angiogenesis is closely associated with the tumour microenvironment and is regulated by a variety of pro-angiogenic factors and/or angiogenic inhibitors.

**Methods:** In the present study, a comprehensive characterization of angiogenesis genes expression was performed in a pan-cancer analysis across the 33 human cancer types. Further, genetic data from several public databases were also used in the current study. An angiogenesis score was assigned to The Cancer Genome Atlas (TCGA) pan-cancer data, with one angiogenesis score as per sample for each tumour.

**Results:** It was found that angiogenesis genes vary across cancer types, and are associated with a number of genomic and immunological features. Further, it was noted that macrophages and iTreg infiltration were generally higher in tumours with high angiogenesis scores, whereas lymphocytes and B cells showed the opposite trend. Notably, NK cells showed significantly different correlations among cancer types. Furthermore, results of the present study showed that a high angiogenesis score was associated with poor survival and aggressive types of cancer in most of the cancer types.

**Conclusion:** In conclusion, the current study evidently showed that the expression of angiogenesis genes is a key feature of tumour biology that has a major impact on prognosis of patient with cancers.

## Introduction

Given that malignant tumours need supplies of oxygen and nutrients to survive and thrive, they require adequate vascularization to access the blood circulation system (Lugano et al., 2020). Previous studies have shown that rapid growth of tumours requires a large supply of nutrients from the blood as compared with dormant tumours. Therefore, it should be noted that initiation of tumour angiogenesis is a major factor for tumor development (Azoitei et al., 2010). Results of a previous clinical trial showed that anti-angiogenic therapy can be successfully used to treat cancer (Hurwitz et al., 2004). Recently, anti-tumour angiogenesis research has evolved from the early non-specific embolisation and severance of tumour vessels to a new level of specific and targeted blockade of tumour vessels (Hida et al., 2008; Mitsuhashi et al., 2015). However, it was found that the anti-angiogenesis therapy only provided a short-term relief and inhibition of tumour growth before resistance is developed (Lugano et al., 2020). Further, emerging evidence shows that angiogenesis and immunosuppression frequently occur simultaneously in response to this crosstalk. Accordingly, strategies combining both anti-angiogenic therapy and immunotherapy have potential to tip the balance of the tumor microenvironment and improve treatment response (Solimando et al., 2020).

The present study provides a comprehensive assessment of the genomic and clinical characteristics of angiogenesis genes in 33 solid tumours. In addition, the angiogenesis score of each patients with cancers was also assessed in the current study. Results of this study found that angiogenesis is correlated with distinct genomic and immunologic tumour characteristics. Moreover, it was found that expression of angiogenesis genes has prognostic and predictive value in outcomes of both patients and their response to immunotherapy. Therefore, the analyses reported in the current study provide the first comprehensive survey of angiogenesis genes expression across 33 cancer types.

## Materials and Methods

### Datasets

**FIGURE 1:**
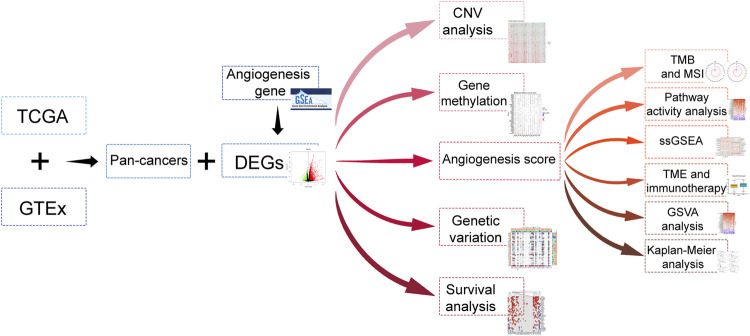
Article flow diagram.

The data of 33 tumors in The Cancer Genome Atlas (TCGA) including the mRNA data, mutation data, and clinical data were collected from UCSC Xena. The data on expression of gene for different tissues were retrieved from GTEx, whereas the angiogenesis relevant data was downloaded from the hallmark gene sets of the msigdb database. The flow of this articles is as presented in Figure 1.

### Identification of Differential Genes

The present study analyzed differential expression of angiogenesis genes in tumour samples (from TCGA database) as compared with that of the normal samples (from TCGA and GTEx database). The “limma” R package was used to identify differentially expressed genes (DEGs), with FDR <0.05, and |log2FC| ≥ 1 as the cut-offs. Next, “ggplot” and “reshape” R packages were used to generate a heatmap to visualize the results obtained.

### Survival Analysis of Angiogenesis Genes

The ‘Survival’ R package was used to conduct univariate Cox regression analysis for angiogenesis genes. Further, the *p*-value and HR-values were then extracted for heatmap presentation. The number of genes was then counted for which Cox analysis was significant in all tumours. Notably, a risk factor was considered if the score was increased by 1, whereas a protective factor was considered if the score was decreased by 1 and the final score was used as the risk score.

### Genetic Correlation and Genetic Variation

In the present study, Pearson’s r was used to explore correlations between different angiogenesis genes and the obtained results were presented using a heatmap. Genetic variation data was downloaded from the cbioportal database including mutations, fusions, amplifications, deletions, and multiple variants. Angiogenesis genes variants were then calculated for each tumour. Finally, mutation of angiogenesis genes in different cancers was explored using Gene Set Cancer Analysis (GSCA) (http://bioinfo.life.hust.edu.cn/GSCA).

### Gene Methylation Analysis and Copy Number Variation Analysis

Previous studies have suggested that DNA hypomethylation promote increased expression of many oncogenes, whereas DNA hypermethylation has also been shown to silence tumour suppressor genes (Wang et al., 2020). In the present study, the mRNA expression and methylation data for the angiogenesis genes were merged using the GSCA, followed by the analysis of the differential methylation. Further, a Student’s *t* test was performed to determine the methylation difference between tumor and normal samples, and the *p*-value was adjusted using the FDR. Notably, FDR ≤0.05 was considered statistically significant. Pearson’s or Spearman’s correlation was then performed to explore the association between methylation and expression levels. Finally, HR for prognostic value of gene methylation status was calculated using Cox regression analysis. The information of copy number variations (CNVs) of the 33 types of tumors from the TCGA database and determined using GISTIC 2.0 software were also totally collected. In addition, the percentage of CNVs and CNVs correlation with mRNA were calculated by Spearman correlation analyses by corrplot package. The CNVs were classified into 2 categories (homozygous and heterozygous), including amplification and deletion, which represents the presence of CNVs on only one chromosome or two chromosomes. It is worth noting that only genes with CNV >5% in cancer were discussed in the present study. The associations between paired mRNA expression and paired CNV percentage samples were explored based on the Person’s product moment correlation coefficient and t-distribution (Schlattl et al., 2011). The statistical significant threshold was set as FDR *p*-value ≤0.05.

### Angiogenesis Score

The angiogenesis score for each sample in the present study was calculated using the “GSVA” R package (Kim et al., 2020). The size of the angiogenesis score in each tumour and explored the differences in angiogenesis score across clinical stages. Data visualization was done using “ggplot” R package (Shatalova et al., 2020). Next, the relationship between the angiogenesis score and prognoses in patients with cancer was explored. Survival estimates were calculated using Kaplan-Meier (KM) and Cox regression models. The “GSVA” R package was used to perform GSVA analysis, with t value >2 and FDR <0.05 as the cut-offs. Notably, the gene set used in this study was MsigDB dataset, HALLMARK pathway of the database. Single-sample gene set enrichment analysis (ssGSEA) was also employed to evaluate the enrichment scores for each sample. Therefore, the present study also evaluated the correlation between angiogenesis score and pathways. Finally, the correlation was visualized using “ggplot” R package (Shatalova et al., 2020).

### Tumor Microenvironments and Immunotherapy

Immune and stromal scores were calculated using the ESTIMATE algorithm with the help of “limma” and “estimate” R packages. The relationship between the angiogenesis score and tumor microenvironment in accordance with a previous study was also assessed to validate the results obtained in the current study (Zeng et al., 2019). Finally, the data was plotted using the “pheatmap” R package. Considering the association of immune infiltration level with survival and prognosis in cancers, the correlation between angiogenesis score and immune infiltration level was hence explored. On the other hand, the CIBERSORT algorithm was used to estimate data on tumor-infiltrating immune cells (Thorsson et al., 2018; Huang et al., 2021). Immune Cell Abundance Identifier and TIMER2 were used to evaluate the correlation of angiogenesis score with immune infiltration across all tumors in the TCGA database. Plots were then performed using “ggplot” R package (Shatalova et al., 2020). The use of immunotherapy in cancer treatment has evolved rapidly and several therapeutic antibodies have reached the clinical practices in recent years (Hultqvist et al., 2017; Wu and Shih, 2018). Therefore, the present study used immunotherapy data to explore the impact of high or low angiogenesis score on the prognosis of immunotherapy patients. Specifically, immunotherapy data was used from IMvigor210 dataset, GSE78220, GSE135222, and GSCA.

### Correlations Between Angiogenesis Score and Immunological Genes

The correlation between immune genes and angiogenesis score was also analyzed in the present study. The analyzed target genes included MHC genes, chemokines, chemokine receptors, and immunosuppressive genes. Recently, tumor mutation burden (TMB) has emerged as a predictive indicator for tumor immunotherapy, which aids in prognostic prediction of immunotherapy in some tumors such as lung cancer, malignant melanoma, and colon cancer (Kelley et al., 2020; Saeed and Salem, 2020). Microsatellite instability (MSI) is a genetic change that has been shown to be closely associated with tumor prognosis (De Palma et al., 2019; Li et al., 2020a). In the present study, the TMB score was calculated using R software and corrected by dividing the total length of the exon. In addition, the MSI scores for all samples were obtained from the somatic mutation data which was downloaded from TCGA database. Finally, Spearman correlation analysis was performed to assess the correlation of angiogenesis score with TMB and MSI scores.

## Results

### Dysregulated Angiogenesis Genes in Various Cancer Types Are Associated With Prognosis

**FIGURE 2:**
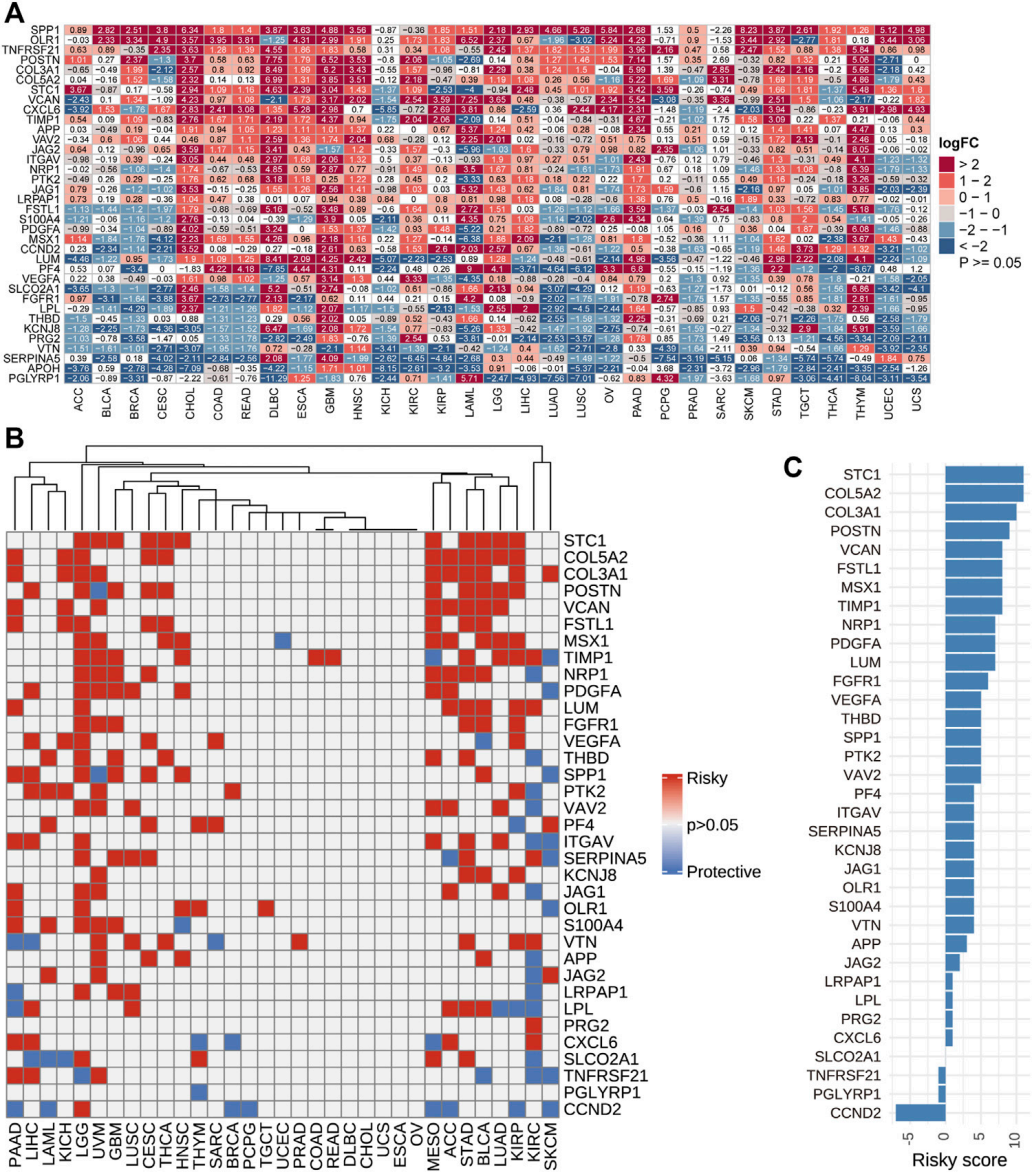
**(A)**: Differential expressions of angiogenesis-related genes. We found the angiogenesis genes were aberrantly expressed in 31 tumor types. **(B)**. Univariate Cox regression analysis of angiogenesis-associated genes in pan-cancer. Grey denotes *p* > 0.05, red represents *p* < 0.05 HR > 1while blue denotes HR < 1 **(C)**. The number of genes with significant cox analysis in all tumors was counted. As a risk factor +1, as a protective factor -1, the final score as risky score.

Using TCGA and GTEx datasets, we evaluated the differential expressions of angiogenesis-related genes between cancers and normal tissues. The angiogenesis genes were aberrantly expressed in 31 tumor types (*p* < 0.05, Figure 2A). We found that angiogenesis genes tended to be significantly downregulated in BRCA (Breast invasive carcinoma) and UCES(Uterine Corpus Endometrial Carcinoma), but upregulated in PAAD (Pancreatic adenocarcinoma), GBM (Glioblastoma multiforme). These findings are consistent with several previous studies (Ardito et al., 2008; Liu et al., 2019). For example, SPP1 was upregulated in most tumor types. SPP1 enhances tumor development and its overexpression is associated with poor prognostic outcomes for melanoma, while its silencing suppresses melanoma cell proliferation, migration, as well as invasion (Komatsu et al., 2012; Deng et al., 2020). These findings imply that dysregulated expressions of angiogenesis-associated genes are involved in cancer initiation and development. Since angiogenesis-related genes play critical roles in cancer metastasis (Firestone and Sundar, 2009), we evaluated the associations between their expression levels and survival outcomes. In at least one cancer type, all angiogenesis-related genes were associated with overall survival outcomes (Figure 2B). In many cancer types, patients with elevated angiogenesis-associated gene levels have significantly poor survival outcomes, compared to those with suppressed levels. Risk scores revealed that most genes were unfavourable for patient prognosis. These findings imply that angiogenesis-related gene levels are associated with prognostic outcomes in many human cancer types, with suppressed levels exhibiting protective effects (Figure 2C).

### Analysis of Angiogenesis Gene Variants and Methylation

The relationship between angiogenesis genes was established to be positively significant (Supplementary Figure S1A), suggesting that these genes could function individually or cooperatively. Notably, BRCA samples exhibited the highest number of variant genes. Genetic variabilities of *STC1* and *LPL* genes were highest, and they were dominated by deletions while gene fusions were dominant among the other gene variants (Supplementary Figure S1B). The development of many tumors has been associated with point mutations and deletions (Agirre et al., 2006). The single nucleotide variation (SNV) was dominated by C > T (50%) (Supplementary Figures S2A-S2B). Supplementary Figure S2C show the frequencies of deleterious mutations in pan-caner. For instance, elevated *VCAN* levels in gastric cancer independently predict poor prognostic outcomes, and patients with *VCAN* mutations have a lower tumor grade, compared to those without (Li et al., 2020b; Li et al., 2020c). Moreover, Supplementary Figure S2D shows survival differences between mutant (deleterious) and wild type in the selected cancers. Aberrant methylation status are correlated with the development of various diseases, including cancer (Shao et al., 2017). Evaluation of the methylation status of angiogenesis-associated genes in this study was aimed at identifying epigenetic regulation. Supplementary Figure S2E shows the differences between tumor and normal samples with regards to gene methylation. In different tumors, the methylations of angiogenesis-associated genes were highly heterogeneous. Relative to hypomethylated genes, there were more hypermethylated genes in BRCA, prostate adenocarcinoma (PRAD), uterine corpus endometrial carcinoma (UCEC), and colon adenocarcinoma (COAD). In addition, in most cancers, *PGLYRP1, KCNJ8,* and *LPL* were hypermethylated while *OLR1*, *VEGFA,* and *SPP1* were hypomethylated (Supplementary Figure S2E). Correlation analysis revealed that mRNA levels of angiogenesis-associated genes were negatively correlated with their methylation levels. However, in PRAD, skin cutaneous melanoma (SKCM), BRCA, liver hepatocellular carcinoma (LIHC), and uveal melanoma (UVM), *PTK1* exhibited positive correlations between methylation and mRNA expression levels (Supplementary Figure S2F). Survival analysis showed that in most cancers, hypermethylations of *PGLYRP1, PTK2, THBD,* and *CCND2* as well as hypomethylations of *VAV2, OLR1,* and *ITGAV* were associated with poor survival outcomes (Supplementary Figure S3). Hypermethylation of CCND2 was associated with female lung cancer and lung adenocarcinoma (Hung et al., 2018). Hypermethylation of CCND2 in lung and breast cancer is a potential biomarker and drug target (Hung et al., 2018).

### CNV of Angiogenesis Genes Are Associated With Prognosis

CNV has been confirmed to have diagnostic, prognostic or therapeutic significances in various types of cancer (Su et al., 2018). In the present study, heterozygous amplifications and deletions were the main CNV types (Supplementary Figure S4A). For instance, CNV percentage analysis revealed that homozygous amplifications of *PTK2* in OV, ESCA, BRCA LIHC, and UVM as well as *CCND2, KCNJ8,* and *OLR1* in TGCT were all greater than 35% (Supplementary Figure S4B). Homozygous deletions of *LPL* and *STC1* in PRAD were all greater than 35% (Supplementary Figure S4B). Heterozygous analysis showed that the amplified gene (*PTGFA*) in TGCT, READ, GBM, and KIRP was greater than 35% in all cases, while *STC1* deletion in OV, LUSC, and LUAD was greater than 35% in all cases (Supplementary Figure S4C). Supplementary Figure S5 shows the differences in survival outcomes between CNV and wild types for selected cancers. These results imply that the CNV of angiogenesis-associated genes mediate their abnormal expressions, suggesting that it plays a significant role in cancer development.

### Associations Among Angiogenesis Scores, Clinical Stage and Patient Outcomes

For the 33 tumors, the highest angiogenesis scores were in PAAD while the lowest were in LAML (Supplementary Figure S6A). The scores in ACC, BLCA, BRCA, COAD, ESCA, LUSC, SKCM, STAD, and UVM varied across clinical stages. As clinical staging increased, so did the angiogenesis scores (Supplementary Figures S6B-J). Supplementary Figures S7A-D shows the associations between angiogenesis scores and pan-cancer overall survival (OS), disease free interval (DFI), progression-free interval (PFI), as well as disease-specific survival (DSS). Notably, HR was evaluated in the Cox proportional hazard models. In most cancers, high angiogenesis scores were associated with poor survival outcomes (Supplementary Figure S8). These results suggest a close association between angiogenesis scores and patient outcomes.

### Pathway Activity Analysis and Enrichment Analysis

**FIGURE 3:**
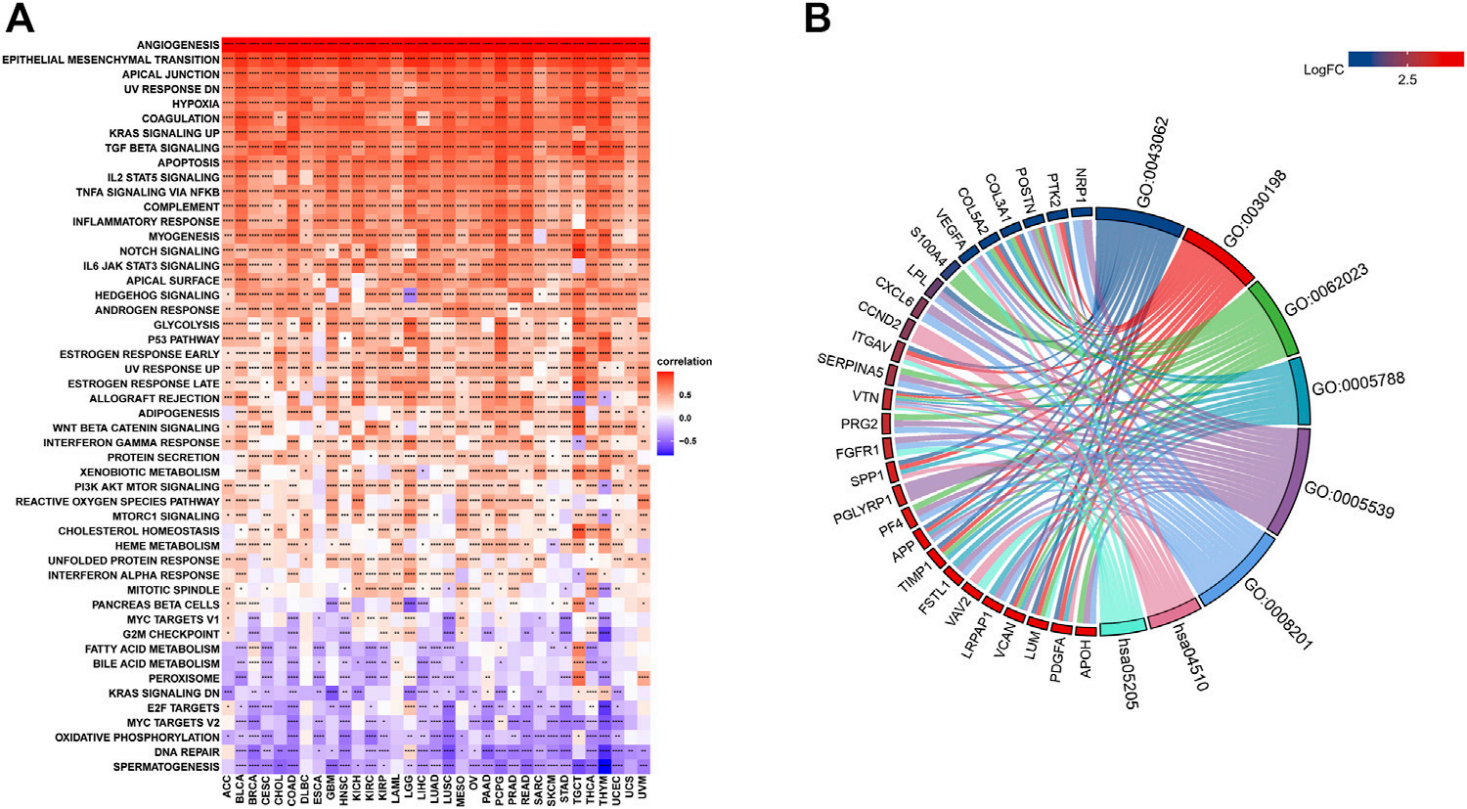
**(A)**. Gene Set Variant Analysis (Red represents positive correlations while blue represents negative correlations. Angiogenesis scores were significantly involved in cancer-related signaling pathways, including Kras signaling, TGF-beta signaling, apoptosis, IL-2 STAT5 signaling, TNF-a signaling via NFK-b, inflammatory responses, IL-6 JAK STAT3 signaling, notch signaling, and the P53 pathway. *** denotes *p* < 0.001, ** denotes *p* < 0.01, while * denotes *p* < 0.05). **(B)**. Enrichment analysis A predominant enrichment in the PI3K-Akt signaling pathway, proteoglycans in cancer, human papillomavirus infections, and the relaxin signaling pathway in BRCA.

The related pathways network indicated that angiogenesis scores were significantly involved in cancer-related signaling pathways, including Kras signaling, TGF-beta signaling, apoptosis, IL-2 STAT5 signaling, TNF-a signaling via NFK-b, inflammatory responses, IL-6 JAK STAT3 signaling, notch signaling, and the P53 pathway (Figure 3A). However, these associations differed among tumors. For instance, in all cancer types, the IL-2 STAT5 signaling pathway was positively correlated with angiogenesis scores, while the DNA repair pathway exhibited the opposite trend. Besides, some pathways, such as those in pancreatic beta cells, exhibited different associations in different tumors. Using breast cancer as an example, enrichment analysis revealed a predominant enrichment in the PI3K-Akt signaling pathway, proteoglycans in cancer, human papillomavirus infections, and the relaxin signaling pathway (Figure 3B).

### High Angiogenesis Scores Are Associated With Hot Tumor Microenvironments

**FIGURE 4:**
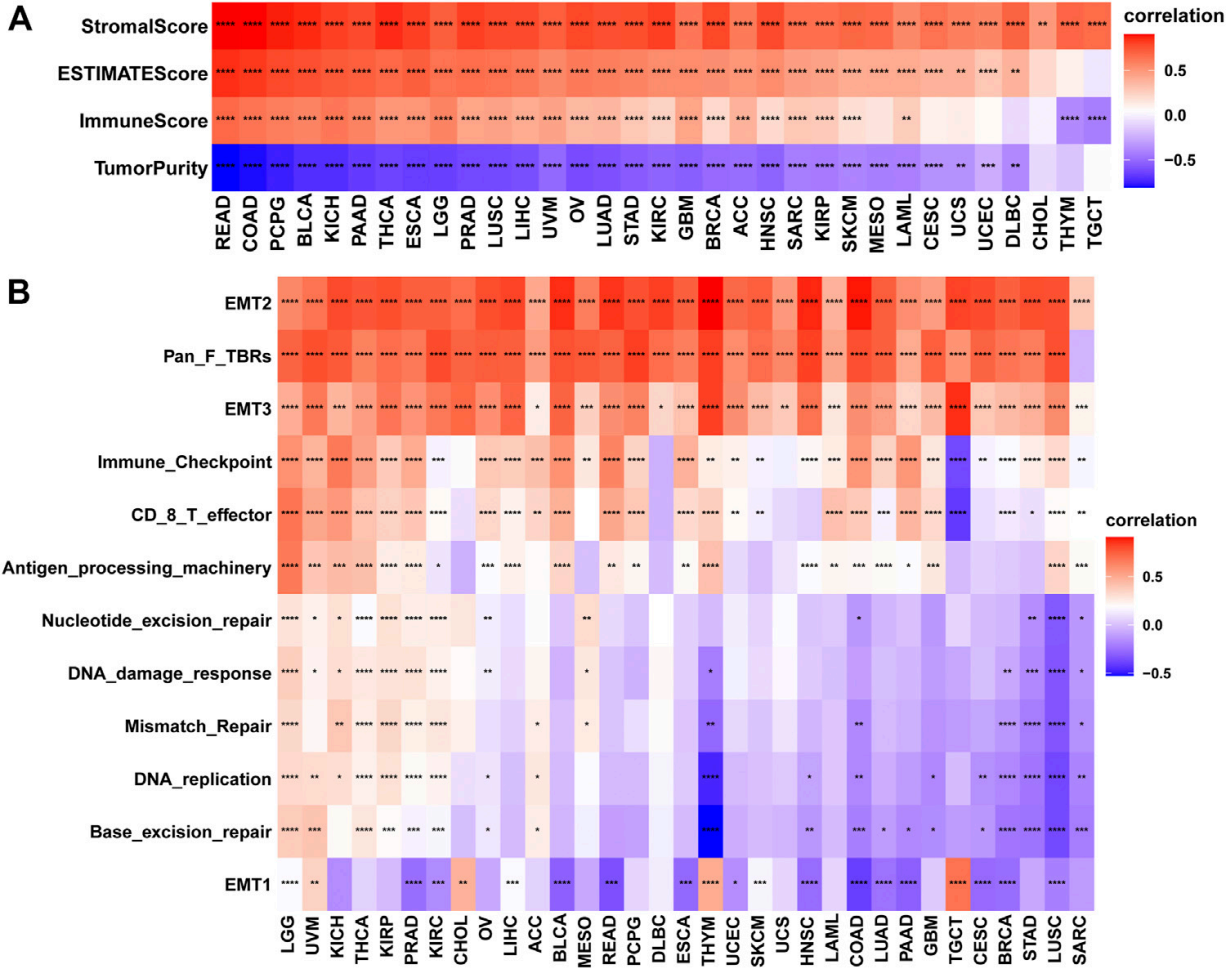
Analysis of the tumor microenvironment. (*** denotes *p* < 0.001, ** denotes *p* < 0.01, while * denotes *p* < 0.05) **(A)**. Correlations between angiogenesis scores and ImmuneScores, Stromalcores, ESTIMATEScores and TumorPurity. **(B)**. Relationship between angiogenesis scores and pathways (Immune-related pathways, Stromal/transfer-related pathways and DNA damage repair-related pathways).

**FIGURE 5:**
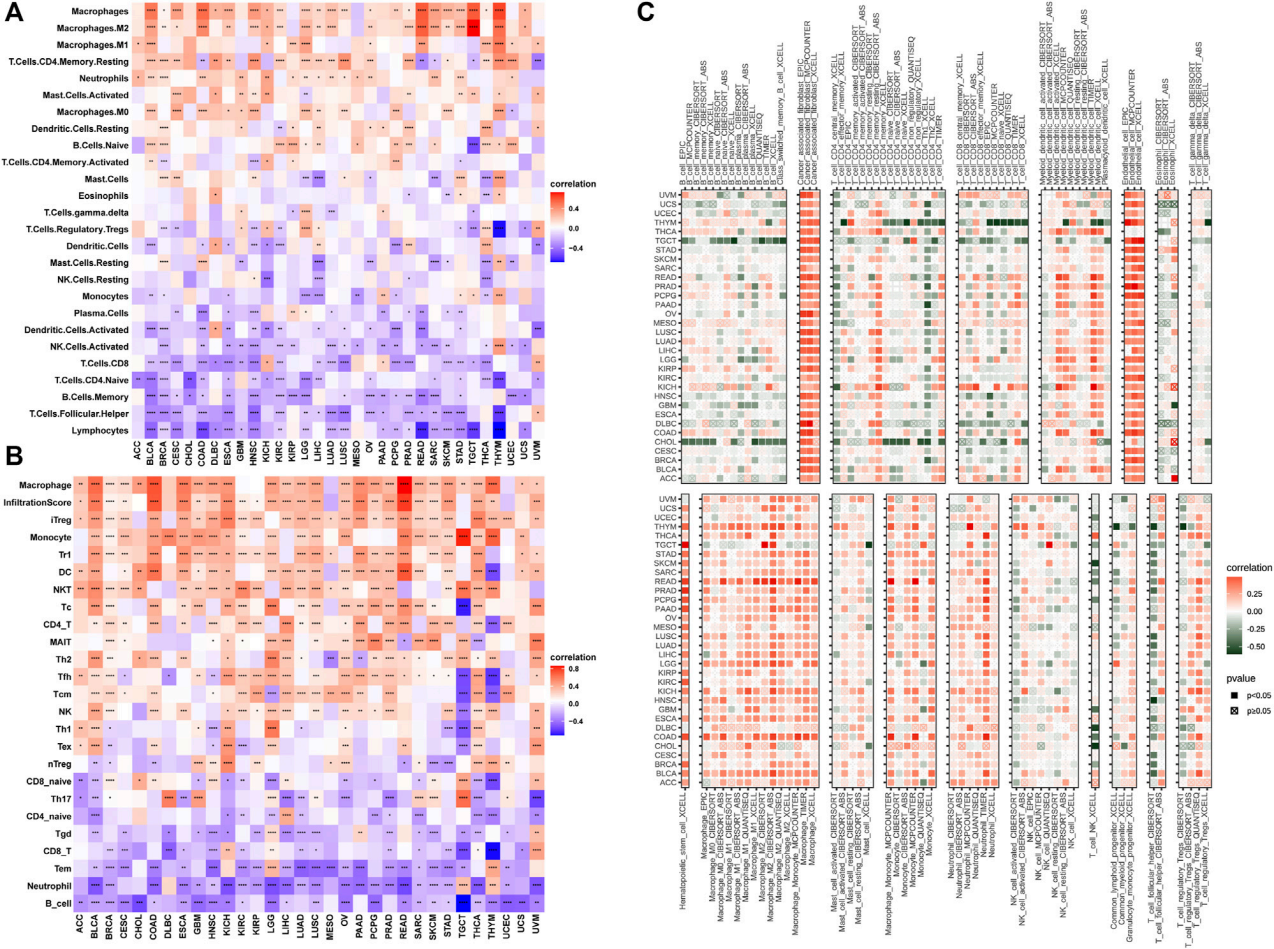
Angiogenesis and immune cell infiltration analysis **(A–C)**: Most of the cancer types showed significant positive associations between angiogenesis scores and infiltration levels of macrophages, NK cells, as well as CD4^+^ T cells).

The tumor microenvironment (TME) modulates tumor progression and treatment efficacy (Blanco‐Fernandez et al., 2021). Therefore, we evaluated the relationship between the angiogenesis score and the TME. Angiogenesis was significantly correlated with the TME (immune and stromal) (Figure 4A) while angiogenesis scores were strongly correlated with immune and stromal/metastasis-related pathways (Figure 4B). Next, the association between angiogenesis scores and infiltration levels of different immune cell types in 33 cancer types were evaluated. Most of the cancer types showed significant positive associations between angiogenesis scores and infiltration levels of macrophages, NK cells, as well as CD4^+^ T cells (Figures 5A,B), implying that angiogenesis scores are associated with a hot TME. For instance, THYM revealed a highly significant positive correlation between angiogenesis scores and infiltration levels of NK cells. In contrast, NK cells exhibited significant negative correlations with angiogenesis scores in TGCT, and weak or no correlations in ACC (Figures 5A–C).

### Angiogenesis Scores Were Associated With Immune-Related Genes in Many Cancer Types

The core function of the immune system is to recognize self and eliminate non-self antigens to maintain normal physiological activities and fight disease (Bai et al., 2020; Forster and Radpour, 2020). This function is largely mediated by the major histocompatibility complex (MHC). The significance of MHC in tumor diagnosis and treatment is being evaluated in immunotherapy (Baba et al., 2007; Marcu et al., 2021). Chemokines are also involved in responses to cancer therapies, therefore, they are potential targets for immunotherapy and chemokine-targeted therapy (Samaniego et al., 2018). We found strong associations between angiogenesis scores and the above related genes (Supplementary Figures S9A-C). Then, we investigated the correlation between angiogenesis scores and expressions of immune checkpoints. In 33 cancer types, the angiogenesis score was positively correlated with expression levels of most of the immune checkpoints (Supplementary Figure S9D). A previous study revealed that TMB is associated with immunotherapeutic responses (Abou Khouzam et al., 2020). We found that angiogenesis scores were significantly correlated with TMB in STAD, HNSC, LUAD, KIRP, LIHC, CHOL, THYM, LAM, and LGG (Supplementary Figure S9E). MSI are repeated DNA sequences (Page and Graham, 2008). Impaired mismatch repair-associated MSI may be a mechanism in gastric cancer development, therefore, its significance is being evaluated by various studies. Our results showed a strong correlation between angiogenesis scores and MSI in BRCA, LUAD, LUSC, STAD, HNSC, and TGCT (Supplementary Figure S9F).

### Angiogenesis Scores Were Correlated With Immunotherapeutic Responses

**FIGURE 6:**
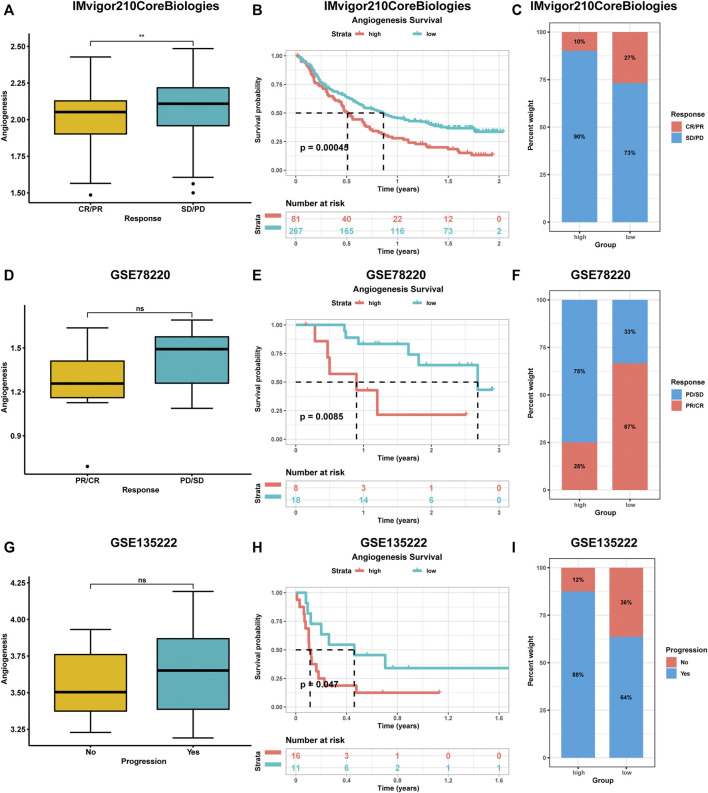
The impact of angiogenesis scores on the prognostic outcome of immunotherapy patients was analysed using multiple datasets **(A-I)**. Patients with high angiogenesis scores exhibited poorer prognostic outcomes after immunotherapy. complete responses (CR), partial responses (PR), progressive disease (PD), stable disease (SD).

During the evaluation of angiogenesis-associated TME characteristics, we observed strong positive correlations between immune checkpoint levels and angiogenesis genes. Due to advances in cancer immunotherapy, we evaluated the expressions of angiogenesis-associated genes in immunotherapy-treated patients. We collected three datasets containing pre-treatment samples and immunotherapeutic data. These datasets had various patient information, including clinical manifestations (complete responses (CR) or partial responses (PR)) or no clinical benefits (progressive disease (PD) or stable disease (SD)). Compared to patients with low angiogenesis scores, patients with high angiogenesis scores exhibited poorer prognostic outcomes after immunotherapy. Higher scores were observed in progressive phase patients (Figure 6).

### Drug Sensitivity Analysis

Currently, surgery and chemotherapy are the main therapeutic strategies for cancer, however, resistance to chemotherapeutic drugs and molecularly targeted therapies is a major barrier to cancer treatment (Wu et al., 2020). Spearman’s correlation analysis showed that drug sensitivity towards YM155, ZG-10, GW843682X, and S−Trityl−L−cysteine correlated with *APP* levels (positive correlation with IC50). However, resistance towards EHT 1864 and lisitinib correlated with expressions of *APOH* (negative correlation with IC50) (Supplementary Figure S10). In conclusion, dysregulated expressions of angiogenesis-associated genes may be involved in resistance to cancer therapies.

### miRNA and lncRNAs Regulation Analysis

Non-coding RNAs (ncRNAs) are involved gene expression regulation. To ascertain whether angiogenesis genes are modulated by some ncRNAs, first, we predicted upstream miRNAs that have the potential to bind angiogenesis-associated genes. Network visualization was achieved using the Cytoscape software (Supplementary Figure S12). Hsa-miR-106a-5p was regulated and was central to most angiogenesis-associated genes. Therefore, in pan-cancer, hsa-miR-106a-5p might be a potential regulatory miRNA of angiogenesis-associated genes. Next, upstream lncRNAs of hsa-miR-106a-5p were predicted using the starbase database. Sixty eight potential lncRNAs were identified. Relationships visualization was performed using the Cytoscape software (Supplementary Figure S12). These results indicate that ncRNAs regulation of angiogenesis-associated genes might be involved in cancer progression.

## Discussion

Angiogenesis, the formation of new blood vessels, is important for tumor progression (Lin et al., 2010). In 1971, Folkman proposed that tumor growth is dependent on angiogenesis and that inhibition of angiogenesis is a potential therapeutic paradigm for solid tumors (Folkman, 1971). To trigger angiogenesis, tumors overexpress various angiogenic factors and cytokines, as well as endogenous angiogenesis enhancers (Folkman, 1996). Various angiogenic factors have been identified, their corresponding inhibitors developed, and their efficacies in cancer treatment demonstrated (Cao, 2010; Cao and Langer, 2010; Cao et al., 2011; Cao, 2014). Therefore, to understand tumorigenesis, and to investigate potential targets for clinical treatment, elucidation of angiogenesis in cancer is necessary.

In this study, we reveal multiple potential mechanisms of angiogenesis in cancer, including common angiogenesis-associated cancer pathways. According to the results of this study, we found a high frequency of CNVs of angiogenesis-associated genes. Moreover, the CNVs were positively correlated with angiogenesis-associated gene expressions, indicating that copy number variations may affect angiogenesis-associated gene expressions, contributing to tumorigenesis. For example, *S100A4* was frequently amplified in PAAD and was associated with poor patient survival outcomes, consistent with findings from previous studies (Matsubara et al., 2005; Li and Bresnick, 2006). Hypermethylated CCND2 was associated with poor survival rates of KICH, implying that hypermethylated CCND2 may be a driver gene for KICH progression. This is in tandem with findings from previous studies that concluded that hypermethylation of *CCND2* is associated with the progression of various cancers (Hung et al., 2018; Ding et al., 2019). However, in this study, there were inconsistencies between methylation levels and prognostic outcomes, therefore, we postulated that in certain contexts, genetic and epigenetic alterations of angiogenesis-associated genes might result in angiogenesis dysfunctions and promote tumorigenesis. Therefore, studies should be conducted to assess this postulate.

The TME is hypoxic and acidic in nature, and in this environment, tumor cells recruit a number of innate immune cells, the most representative of which are tumor-associated macrophages (TAMs), neutrophils, myeloid-derived suppressor cells (MDSC) and natural killer cells (NK) (Hamaguchi et al., 2020). TAMs are involved in every step of tumor angiogenesis (Komohara and Takeya, 2017). In early stages of tumor development, neutrophils play a key role in promoting angiogenesis. Neutrophils can release MMP-9 to activate endothelial cell growth signals and to promote angiogenesis (Eyileten et al., 2016). They also secrete myeloperoxidase (MPO), which is important for macrophage recruitment and platelet activation (Heslop et al., 2010). In addition, reduction in neutrophil counts significantly inhibits the link between VEGF and its receptors (Li et al., 2012).

Despite its significance in tumor therapy, immunotherapeutic responses are suboptimal, which may be attributed to immunosuppressive tumor microenvironments (Chan et al., 2020). The tumor vasculature carries essential nutrients and oxygen to the tumor tissue and plays an important role in the growth as well as progression of malignant tumors. Abnormal blood vessels can form a physical barrier that limits immunotherapeutic efficacies (Jain, 2005). Abnormal tumor vasculature may inhibit immunotherapeutic effectiveness through hypoxia and development of an acidic microenvironment, which enhances immunosuppression (Jain, 2005). In addition, it can induce a decrease in micro-environmental pH, affecting immune cell functions, therefore, anti-tumor immune cells, such as T lymphocytes and NK cells can become unresponsive in acidic environments and subsequently, undergo apoptosis (Huber et al., 2017). In contrast, immunosuppressive components (such as myeloid and Treg cells) enhance tumor growth in acidic environments (Huber et al., 2017). The combination of anti-angiogenic therapy and immunotherapy has improved prognostic outcomes for patients with different cancer types, including liver, kidney and breast cancers (Rini et al., 2019; Finn et al., 2020; Liu et al., 2020), resulting in improved PFS and OS. This combination provides additional clinical options for patients with liver metastases and positive driver genes (Finn et al., 2020). In addition, this combination has not increased toxicity but has an increased efficacy, while the overall safety profile is manageable.

Drug sensitivity analyses were performed to identify potential drugs that can modulate angiogenesis-associated genes. For instance, a positive correlation was found between *APP* and YM155, ZG-10, GW843682X, and S−Trityl−L−cysteine, suggesting that patients with elevated *APP* gene expressions may be resistant to these drugs. Thus, we postulate that targeting angiogenesis-associated genes may be an effective anticancer treatment approach. Our findings, which revealed variations in angiogenesis at all regulation levels, elucidate on regulation of angiogenesis-associated genes in tumors. These variations may in turn lead to differences in drug efficacies, treatment responses and patient survival outcomes. However, more studies should comprehensively investigate cancer heterogeneity and individual-based treatment approaches.

## Conclusion

This study provides the first comprehensive description of angiogenesis-associated gene expressions in various tumor types. Moreover, we systematically investigated the impact of changes in expressions of all angiogenesis-associated genes on clinical outcomes of various cancer types. Angiogenesis-associated gene expressions were correlated with different genomic and immunological tumor characteristics, implying that they have prognostic values in both immunotherapeutic and standard settings.

## Data Availability

The datasets presented in this study can be found in online repositories. The names of the repository/repositories and accession number(s) can be found in the article/Supplementary Material.

## Abbreviations

TCGA, The Cancer Genome Atlas; DEGs, Differentially expressed genes; OS, Overall survival; FDR, False discovery rate; GO, Gene Ontology; KEGG, Kyoto Encyclopedia of Genes and Genomes; HR, Hazard ratio; CI: Confidence interval; See.
