# Supplementary figures and images for: Association of Angiogenesis Gene Expression With Cancer Prognosis and Immunotherapy Efficacy
Source: Front Cell Dev Biol. 2022 Jan 26;10:805507. doi: 10.3389/fcell.2022.805507 (PMC8826089; doi:10.3389/fcell.2022.805507)

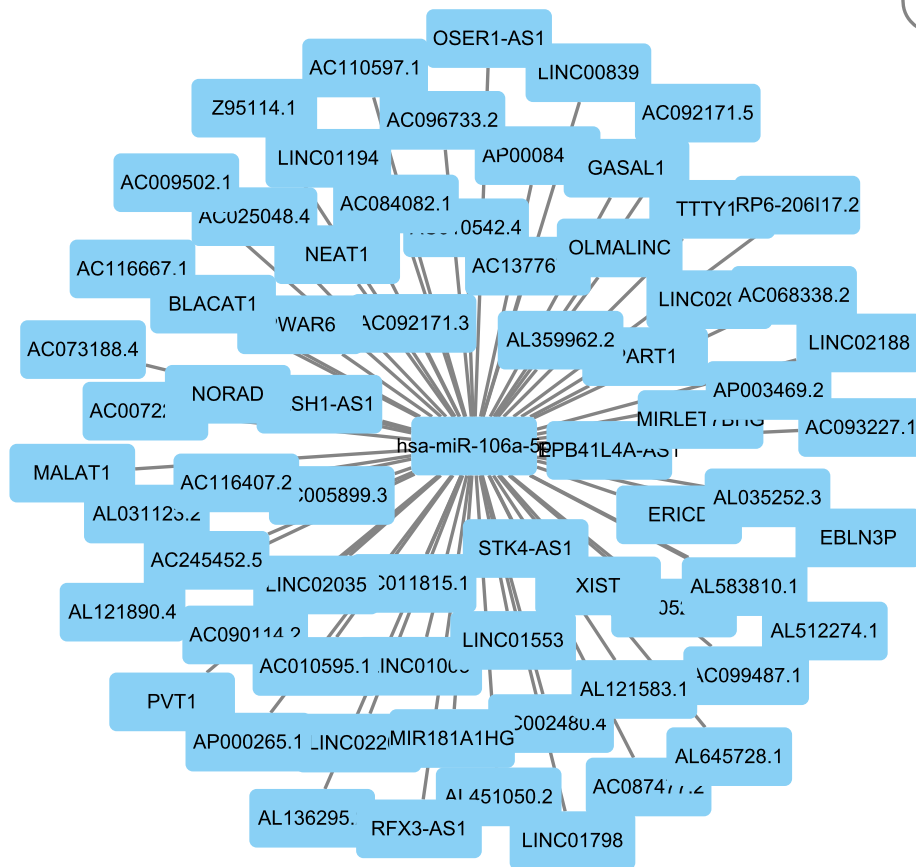

Supplement: Supplementary file 1 [file DataSheet2.PDF]

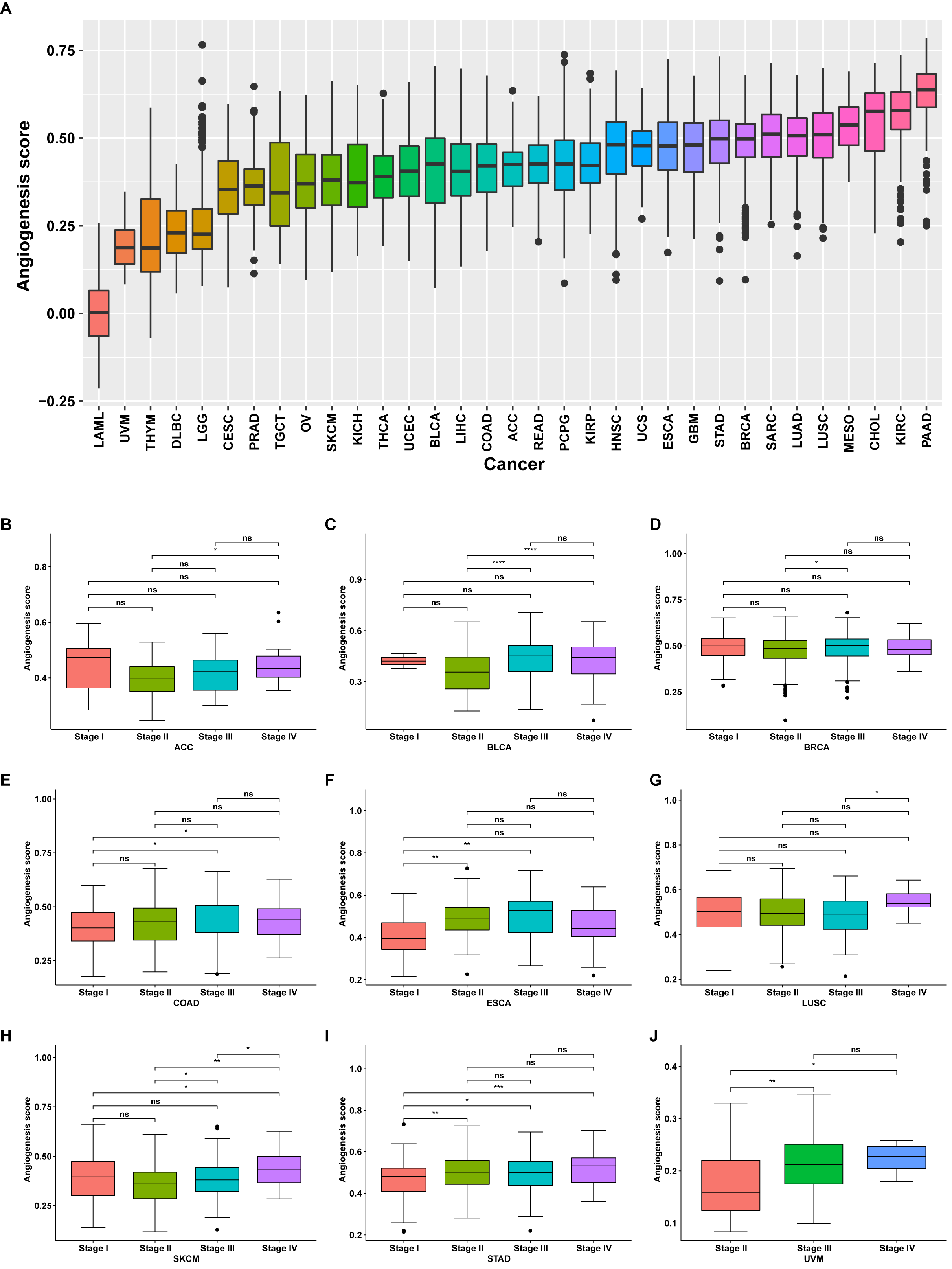

Supplement: Supplementary file 2 [file Image6.TIF]

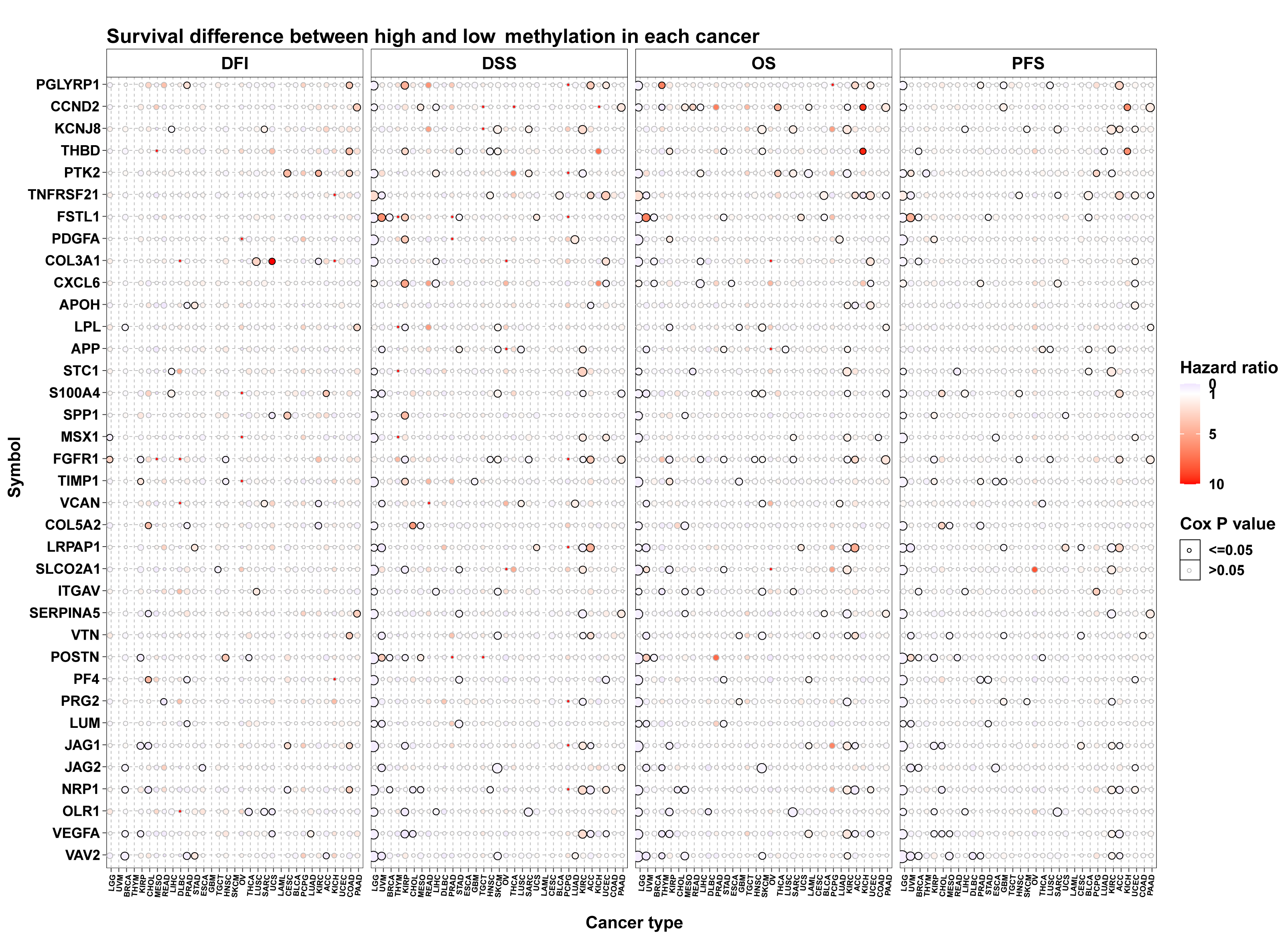

Supplement: Supplementary file 3 [file Image3.TIF]

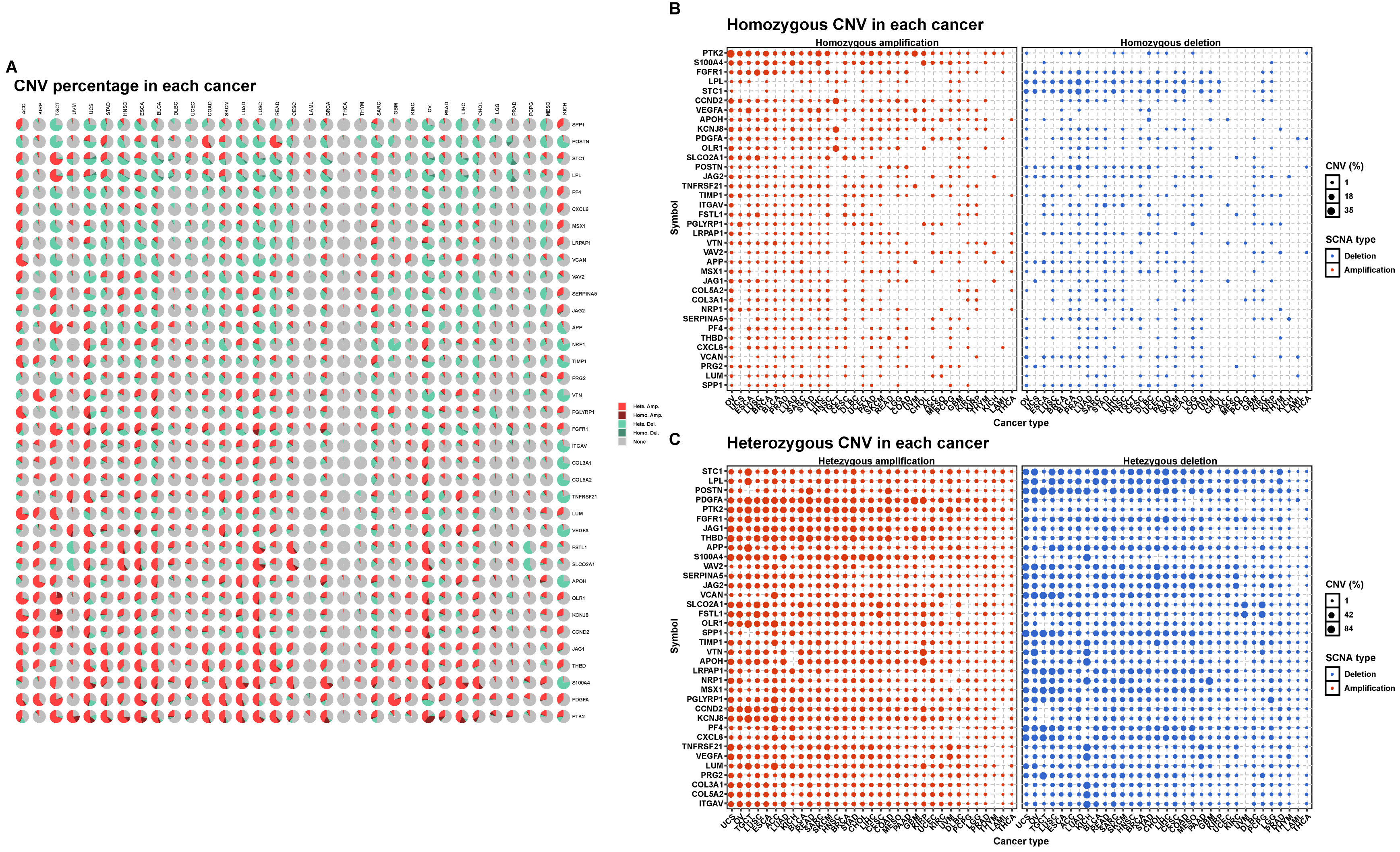

Supplement: Supplementary file 4 [file Image4.TIF]

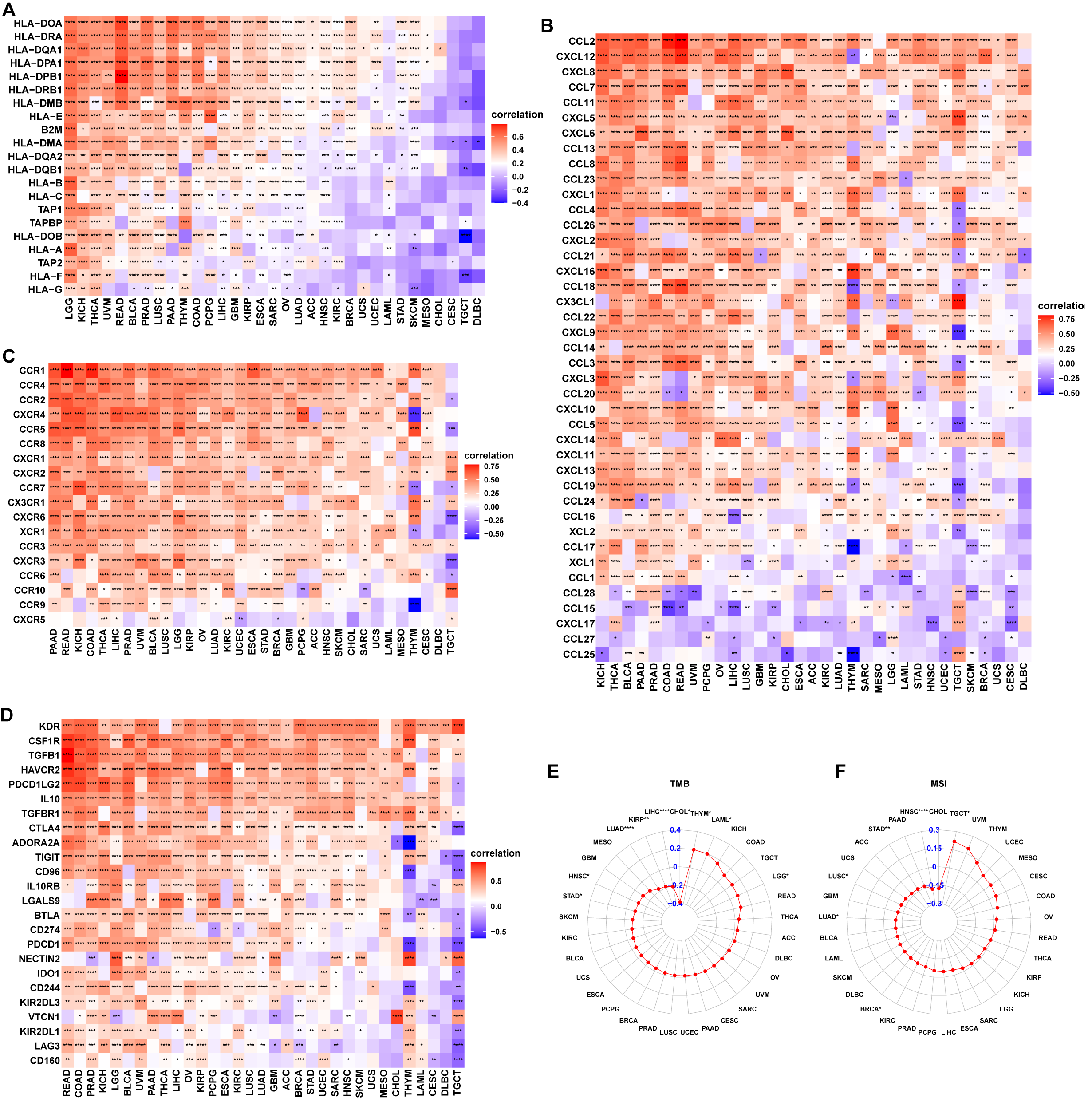

Supplement: Supplementary file 5 [file Image9.TIF]

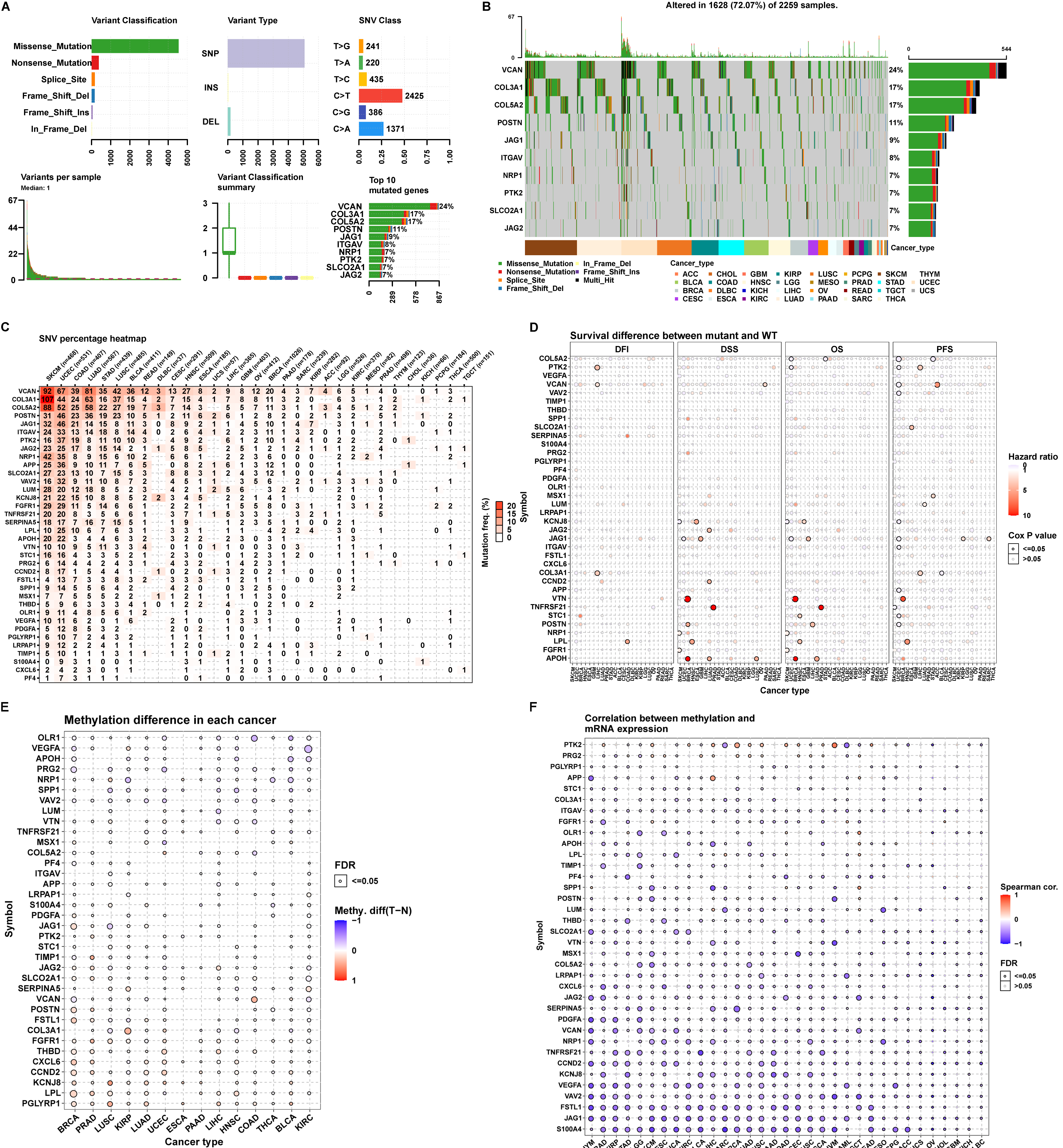

Supplement: Supplementary file 6 [file Image2.TIF]

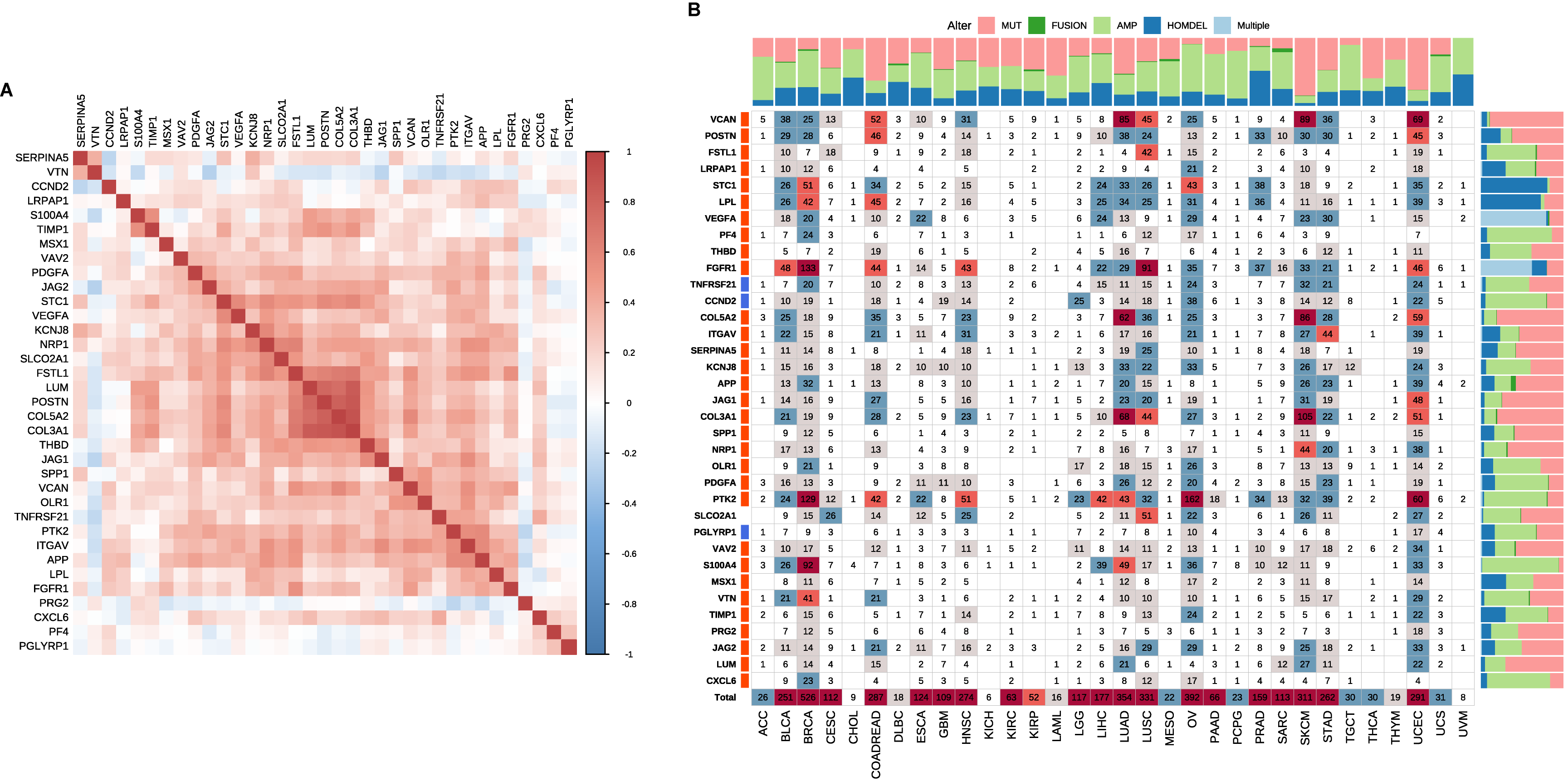

Supplement: Supplementary file 7 [file Image1.TIF]

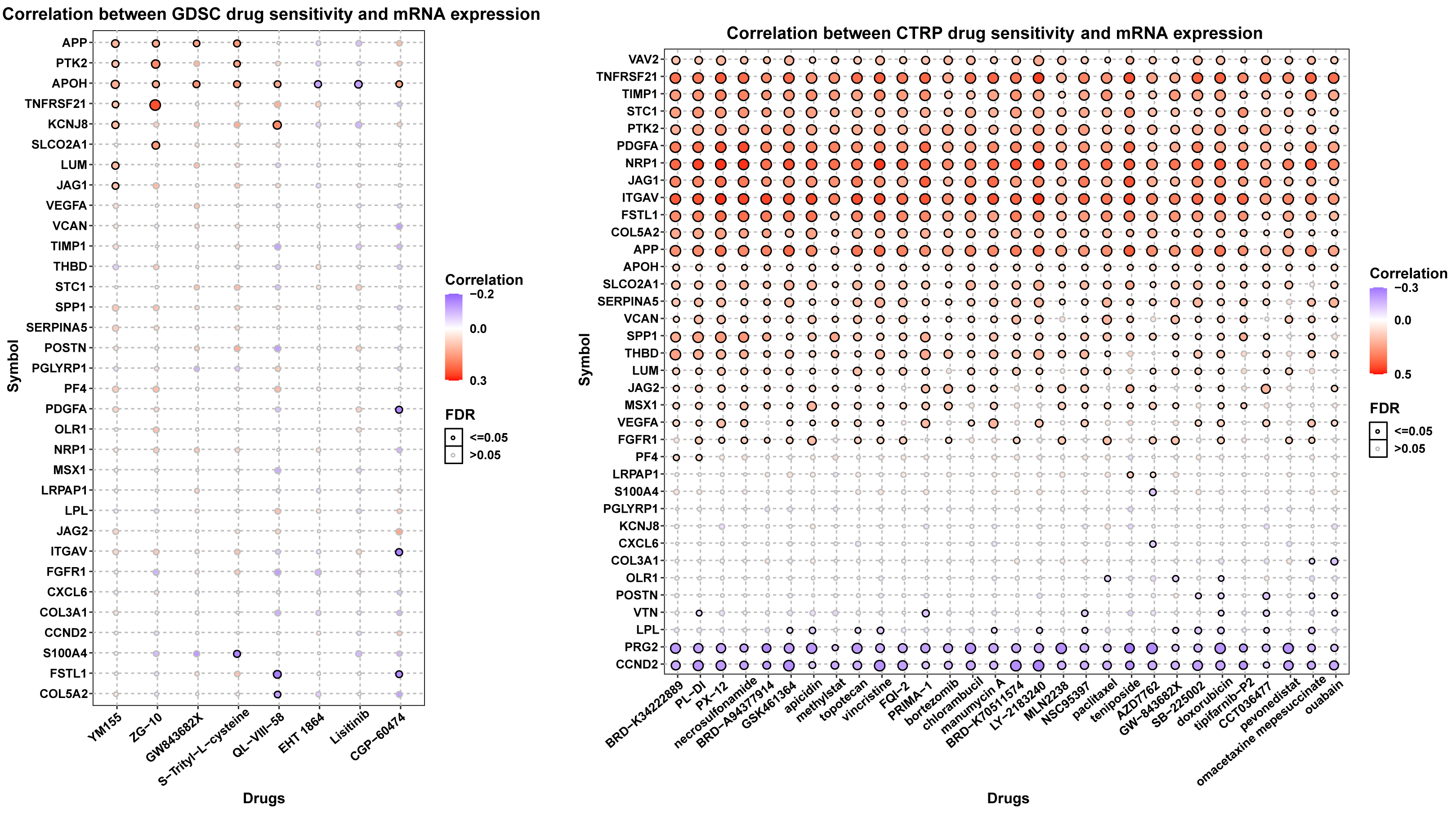

Supplement: Supplementary file 8 [file Image10.TIF]

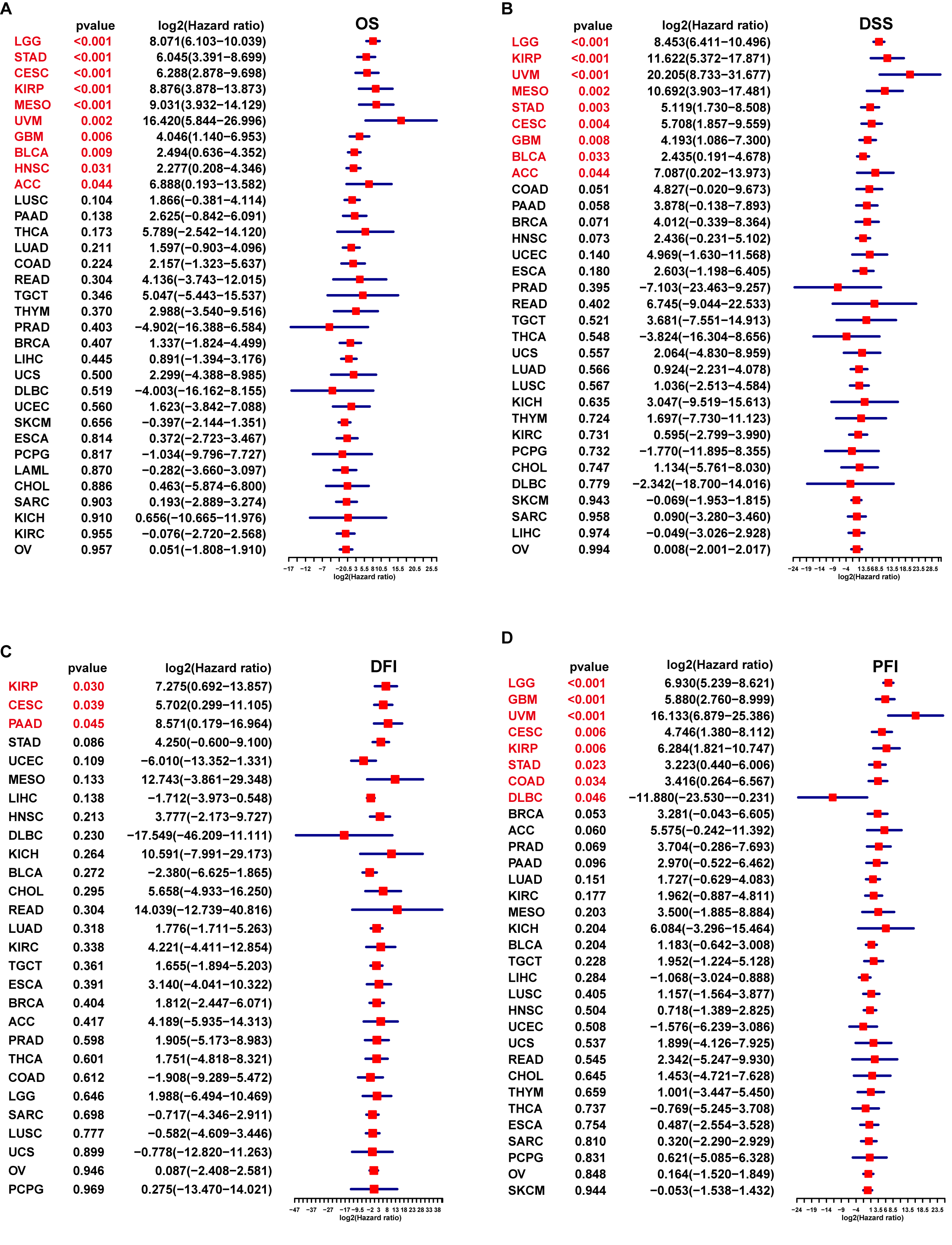

Supplement: Supplementary file 9 [file Image7.TIF]

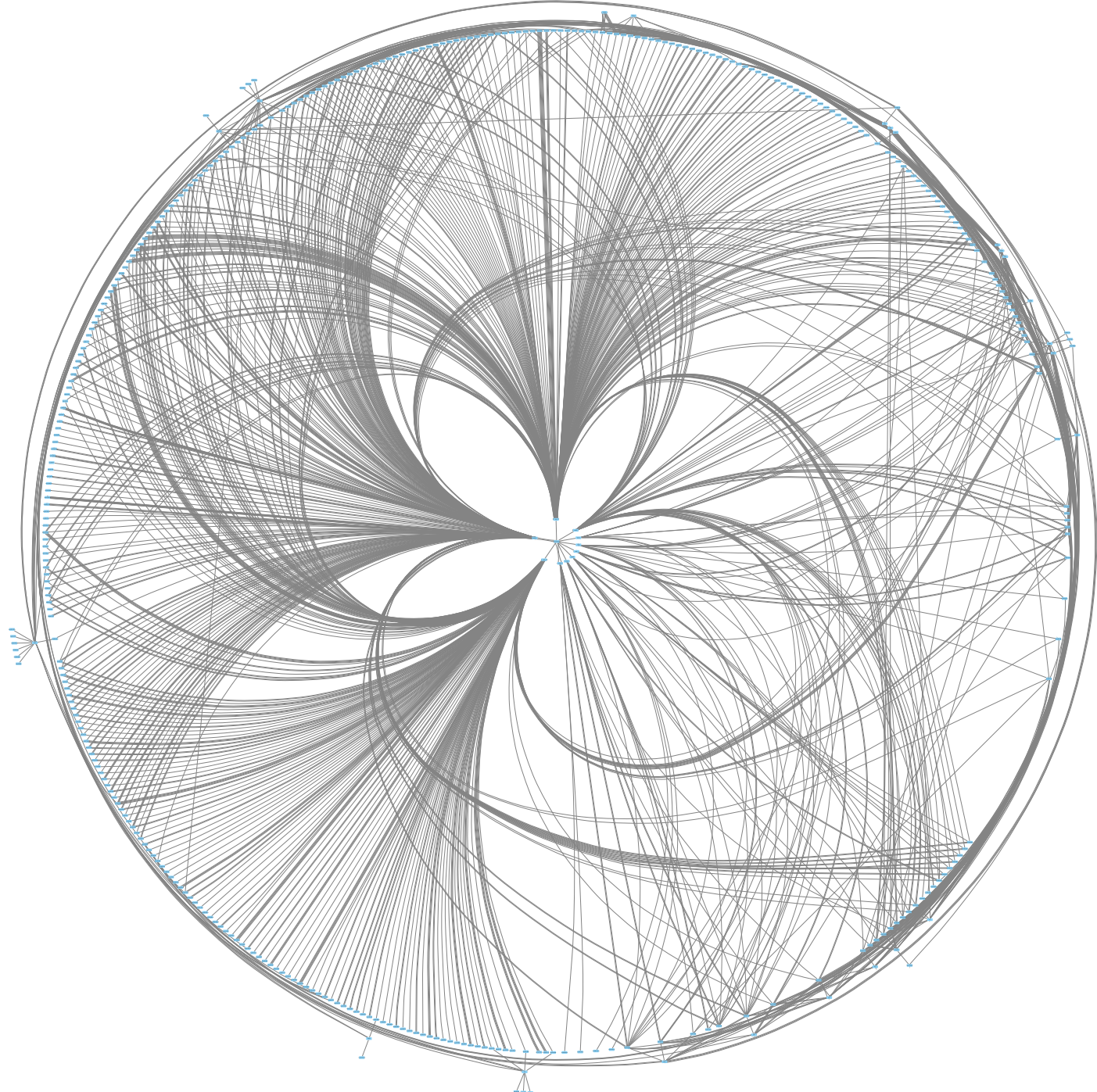

Supplement: Supplementary file 10 [file DataSheet1.PDF]

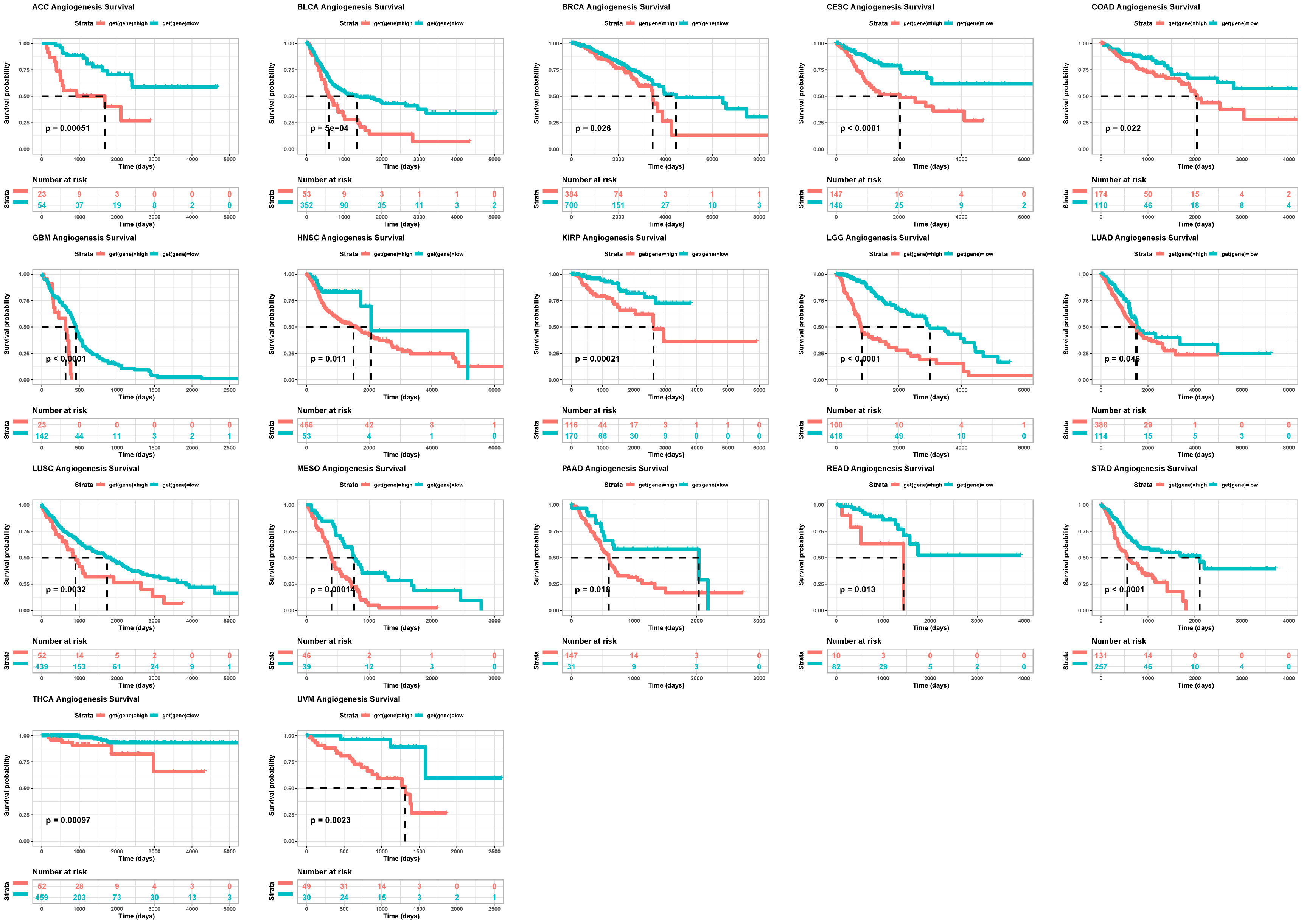

Supplement: Supplementary file 11 [file Image8.TIF]

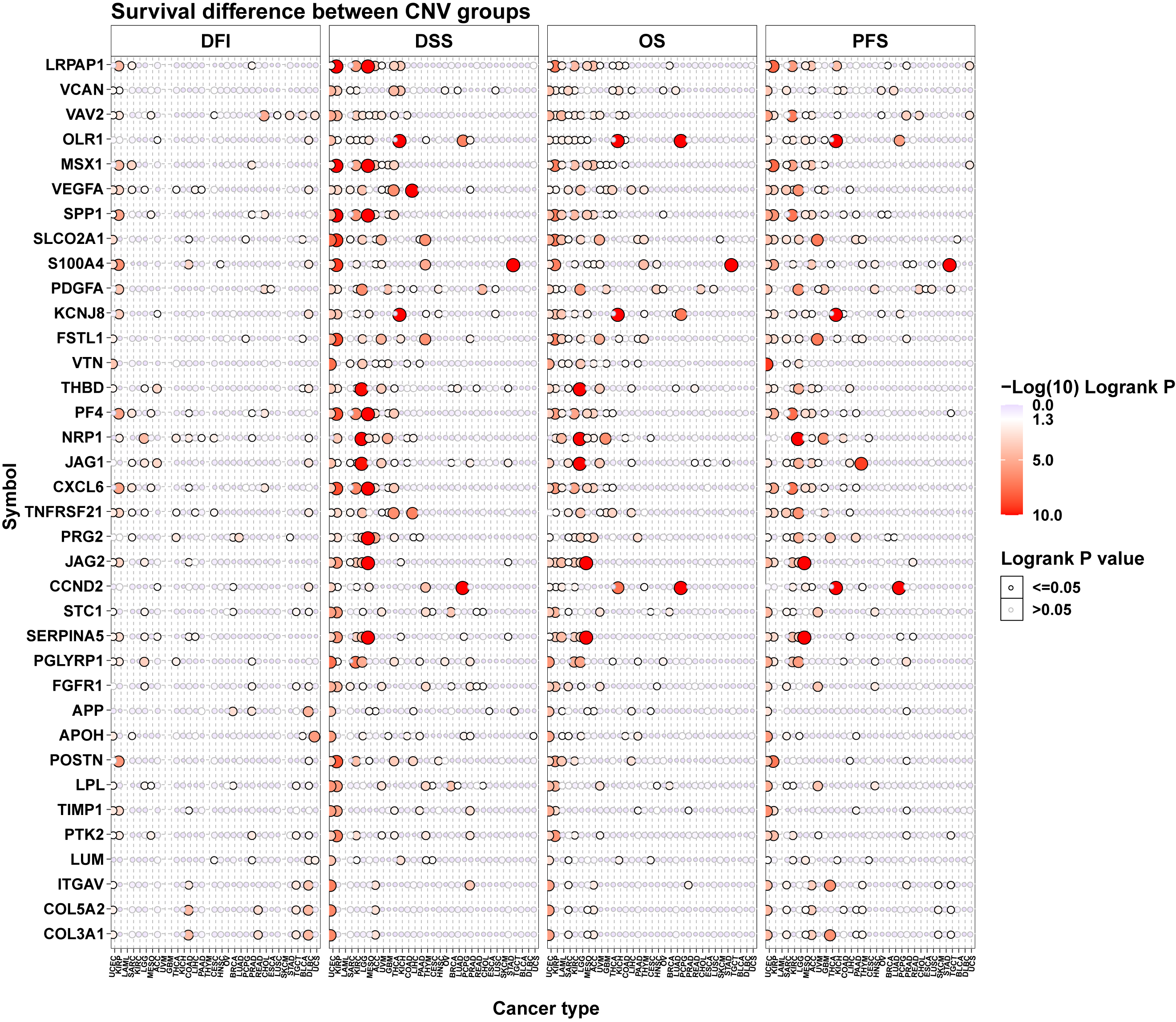

Supplement: Supplementary file 12 [file Image5.TIF]
